# Delamination technique together with longitudinal incisions for treatment of Chiari I/syringomyelia complex: a prospective clinical study

**DOI:** 10.1186/1743-8454-6-7

**Published:** 2009-06-22

**Authors:** Kadir Kotil, Tuğrul Ton, Rabia Tari, Yildiray Savas

**Affiliations:** 1 Department of Neurosurgery, Haseki Educational and Research Hospital, Hasan Ali yücel sok.Senil apt. 36/14 Çiftehavuzlar Kadikoy, Istanbul, 34728 Turkey; 2 Department of Radiology, Haseki Educational and Research Hospital, Hasan Ali Yücel sok.Senil apt. 36/14 Çiftehavuzlar Kadikoy, Istanbul, 34728 Turkey

## Abstract

**Background:**

Treatment modalities in Chiari malformation type 1(CMI) accompanied by syringomyelia have not yet been standardized. Pathologies such as a small posterior fossa and thickened dura mater have been discussed previously. Various techniques have been explored to enlarge the foramen magnum and to expand the dura. The aim of this clinical study was to explore a new technique of excision of the external dura accompanied by widening the cisterna magna and making longitudinal incisions in the internal dura, without disturbing the arachnoid.

**Methods:**

Ten patients with CMI and syringomyelia, operated between 2004 and 2006, formed this prospective series. All cases underwent foramen magnum decompression of 3 × 3 cm area with C1–C2 (partial) laminectomy, resection of foramen magnum fibrous band, excision of external dura, delamination and widening of internal dura with longitudinal incisions.

**Results:**

Patients were aged between 25 and 58 years and occipital headache was the most common complaint. The mean duration of preoperative symptoms was 4 years and the follow-up time was 25 months. Clinical progression was halted for all patients; eight patients completely recovered and two reported no change. In one patient, there was a transient cerebrospinal fluid (CSF) fistula that was treated with tissue adhesive. While syringomyelia persisted radiologically with radiological stability in five patients; for three patients the syringomyelic cavity decreased in size, and for the remaining two it regressed completely.

**Conclusion:**

Removal of the fibrous band and the outer dural layer, at level of foramen magnum, together with the incision of inner dural layer appears to be good technique in adult CMI patients. The advantages are short operation time, no need for duraplasty, sufficient posterior fossa decompression, absence of CSF fistulas as a result of extra arachnoidal surgery, and short duration of hospitalization. Hence this surgical technique has advantages compared to other techniques.

## Background

Sixty six years after Olivier d'Angers first used the term syringomyelia in 1824, a word that means '*cavity *in the spinal cord', H. Chiari described the Chiari malformation, and since Chiari more than a half-century has passed. The etiology and treatment modalities of Chiari malformation continue to be discussed. There are four subtypes of this syndrome of which Chiari malformation Type I (CMI) is the most common. Chiari malformation results in a pressure gradient between the cerebrospinal fluid (CSF) compartments when the cerebellar tonsils descend 3–5 mm or more below the foramen magnum [1-3]. This difference in pressure results in hydrocephaly above the level and syringomyelia below the level. The tonsillary herniation may be symptomatic or not and not every case of CMI is accompanied by syringomyelia. Where syringomyelia exists as in 30%–70% of cases, treatment is required [4,5]. While surgical therapies in CMI focus on posterior fossa decompression, surgical treatment alternatives for syringomyelia vary widely [3,5-11]. Suboccipital craniectomy [3,7], tonsil resection [9], ventriculo-subarachnoid shunt [11], syringo-subarachnoid shunt [8,11], plugging obex [12], syringo-peritoneal shunt [3,13] and syringostomy [14] are the most common ones. In the last decade, widening or decompression of the foramen magnum by microincisions alone [15], and extra-arachnoidal studies have accelerated and started to focus on splitting the outer leaf of dura without duraplasty [16,17]. Because complications due to CSF leakage are avoided and operation time is decreased, dural splitting (delamination) is widely preferable and easy to perform [17].

In our study, using the extra-arachnoidal approach, opening the foramen magnum and the C 1 and C2 laminae, the outer layer of the dura mater was reflected from 3 cm above to 3 cm below the foramen magnum. The second step provided the decompression of the cisterna magna by making longitudinal incisions in the internal dura. This is the first prospective study about this subject using this specific surgical technique.

## Methods

### Patients

Between 2004 and 2006, surgery was performed on ten cases with CMI and syringomyelia and the patients were studied prospectively. Following the approval of the local ethics committee, written informed consent was obtained from all of the participants. All clinical symptoms and findings were recorded preoperatively and postoperatively. Neurological examinations of each patient were recorded in detail according to scoring system defined by Asgari *et al *[18]. All cases underwent surgery as described below and were followed up clinically and radiologically for 25 months.

### Surgical technique

**Figure 1:**
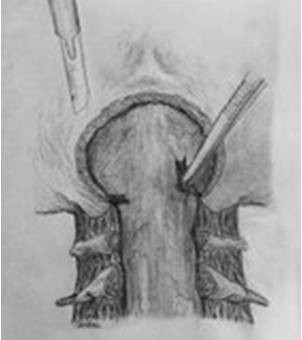
**Illustration of the surgery for the treatment of Chiari I with syringomyelia**. The posterior fýbrous band in the foramen magnum is removed by surgical lancet.

**Figure 2:**
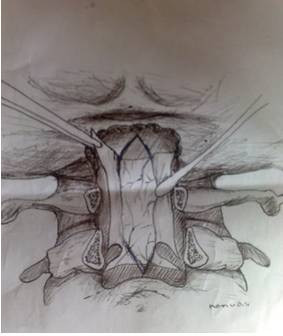
**Illustration showing resection of external dura posterior fossa decompression leaving the internal dura intact**.

**Figure 3:**
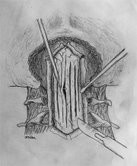
**Illustration of the longitudinal incisions made in the internal dura after the delamination and separation from the external dura with a hook**.

All patients underwent an identical surgical procedure in the prone position. A skin incision was performed using the classical line from the inion to C5 and the muscles were bilaterally opened. When the occiput and cervical region were clearly visible, a craniectomy was made to decompress the foramen magnum over an area 3 × 3 cm using a high speed drill. A total laminectomy to C1 and a partial laminectomy to C2 was performed. The posterior fýbrous band in the foramen magnum was removed (Figure 1). Starting from the craniectomy area in foramen magnum to C2, the outer layer of dura mater was reflected using the dural splitting technique (Figure 2). Subsequently we made longitudinal incisions 3 mm apart in the internal dura from the craniectomy area to C2, using a hook or surgical lancet (Figure 3). The dura mater has two layers at the level of the craniocervical junction. The dura mater gets thinner while passing through foramen magnum to the spinal canal and it is harder to distinguish the two layers. In our series, dura splitting and incisions down to the 2^nd ^cervical vertebra were sufficient because this was the extent of the tonsillar descent in these patients. In addition, a thick constricting dural fibrous band is usually found between the C1 arch and foramen magnum. The outer dural layer is thicker than the internal layer. The dissection and removal of the outer layer is very easy. However incision of the inner layer for the expansion must be done with a hook to avoid damaging the arachnoid membrane and we took great care not to disturb the arachnoid. The mean operation time was 95 min.

### Neurological assessment

The patients were evaluated both before and after the surgery. Reflex change, atrophy, pain and temperature dissociation, paresthesia were all recorded on the 1st day, 1st week, 2nd month, 6th month and 2nd year after surgery. The neurological status was graded according to scoring system defined by Asgari *et al *[18]:

Grade 1: mild (neck pain and headache).

Grade 2: moderate (spinal cord signs exist but function is sufficient). Patients have no myelopathy such as Hoffman's or Babinski signs.

Grade 3: severe (assistance required because unable to use extremities). Patients have myelopathy with Hoffman's and Babinski signs and are unable to use the fingers.

### Radiological assessment

All cases were followed up post-operatively with MRI on day 1, at 2 months, and 2 years. Posterior fossa volumetric values were evaluated from preoperative and postoperative MR images obtained in all cases using the Cavalieri method [19]. Syrinx cavity volumes and regressions, tonsillar ectopy measurements and cisterna magna preoperative and postoperative measurements were all noted.

## Results

### Patients

The mean age was 44.4 years (25 to 58 years), 4 were male and 6 female. The mean duration of preoperative symptoms was 4 years (10 months to 9 years). The most common symptoms were pain, sensory disturbances and loss of sensation (Table 1). In all cases, the presenting symptoms were occipital pain which radiated to the neck and both the upper extremities. The most common neurologic sign was a sensory disturbance of the upper extremities. We have not noticed any significant relation between the amount of tonsil descent into the spinal canal and the syringomyelia. Blood loss was less than 100 ml and the mean operation time was 2 h. In one patient there was a transient CSF fistula that was treated with tissue adhesive.

**Table 1:** Symptoms and signs of ten patients with Chiari malformation Type I and syringomyelia.

**Symptoms and signs**	**Number**	**Percentage %**
Sensory disturbances(upper extremity)	9	90
Pain radiating from head and neck to arms	8	80
Motor weakness(upper extremity)	6	60
Loss of temperature and pain sensation	8	80
Pyramidal tract signs	5	50
Vertigo	6	60
Cerebellar signs	3	30

### Neurological status

Clinical progression was halted for all patients; 8 patients completely recovered and 2 reported no change (Table 2). This effect was present when neurological testing was performed at 6 months after surgery and was unchanged when tested again at 24 months.

**Table 2:** Neurological status of ten patients with Chiari malformation Type I and syringomyelia scored with the Asgari scale before and after the surgery.

**Grade**	**Number of cases**		
	
	Preoperative	Postoperative (6 months)	Postoperative (24 months)
Grade1	8	0	0

Grade 2	2	2	2

Grade 3	0	0	0

### Radiological results

**Figure 4:**
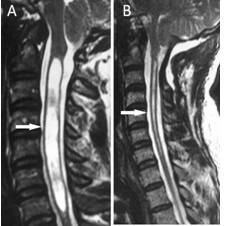
**T_2_-weighted MR images of a patient with Chiari Type I and syringomyelia in the cervical region**. A: The preoperative sagittal image. The syringomyelic cavity is the pale area in the center of the spinal cord (arrow) B: postoperative sagittal image at 12 months after surgery showing the syrinx cavity has decreased in size (arrow). The new iatrogenic cisterna magna is demonstrated.

**Figure 5:**
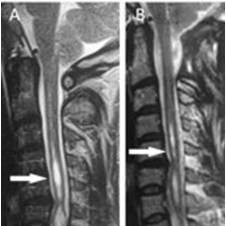
**T_2_-weighted MR images of a patient with Chiari Type I and syringomyelia (arrow)**. A: Preoperative sagittal MRI scan. B: Postoperative image at 15 months after surgery showing a decrease in the size of the syrinx (arrow).

All cases were followed up radiologically for 25 months and the results were recorded. All cases underwent MRI (Figures 4 and 5). Radiological evaluation of the syrinx cavity with MRI and follow-up time is presented in table 3. The syrinx persisted unchanged in five patients; for three patients the syringomyelic cavity was reduced, and for the remaining two patients it regressed completely. No volumetric enlargement of the posterior fossa was detected in our subjects. However, strong and thickened fibro-dural bands at the level of foramen magnum were detected in all cases.

**Table 3:** Radiological and clinical evaluation of the syrinx cavity with MRI of the patients and follow-up time.

Number of cases (10)	Stabilised (unchanged)	Limited regression	Significant regression	Follow-up time (mean & range)
Radiological change	5 (50%)	3 (30%)	2 (20%)	25 mo(13–30)

Clinically improved	2 (20%)	-	8(80%)	25 mo (13–30)

### Complications

No major complications were observed. As a minor complication, in only one case (10%), a CSF fistula and wound infection occurred. This resulted from using a surgical lancet (blade) to make the incision after splitting the external dura; for the remaining of cases, the internal dura was opened with a round edged dural hook.

## Discussion

There are several theories regarding the origin of CMI [2,19-26]. Recently Marin-Padilla has suggested a new theory of partial insufficiency at the time of the mesodermal closure by proposing that the cerebellar tonsils, which pass from a narrow corridor into the spinal canal, impede CSF circulation [27] and many different surgical techniques have been used in the past. This study has shown that surgery consisting of removal of the fibrous band and the outer dural layer, at level of foramen magnum, together with the incision of inner dural layer had a good outcome in a group of ten patients with CMI and syringomyelia.

Techniques such as opening the dura and treating arachnoid scarring have been used before [5].

It is known that compared to the normal population, patients with syringomyelia and CMI have a smaller posterior fossa and cisterna magna [28-31]. However, the importance of dural occlusions and dural bands at the level of the foramen magnum in syringomyelia has not previously been described [19-22]. Therefore, for this pathology it has been suggested that the surgical focus should be on the dura without touching the arachnoid [12,24,32,33].

In a study by Matsumato and Symon, surgery using duraplasty gave better results than surgery without duraplasty [9]. Isu *et al*., in their article published in1993, focused on widening the cisterna magna after foramen magnum decompression using the extradural approach of splitting the outer layer (delamination), and the overall outcome of patients was very satisfactory [16]. Furthermore, Gambardella *et al*, have studied indirect duraplasty by using transverse microincisions, with an extradural approach without delamination and have published positive results [15]. These previous studies suggest that foramen magnum decompression alone is not sufficiently effective without dural delamination. However, duraplasty has disadvantages in that the long processing time can result in a mixture of blood components in the CSF and in CSF fistulas. Some disadvantages such as systemic complications, high rate of CSF fistulas and long hospitalization periods have been reported in patients who underwent duraplasty and arachnoid dissection [34-36].

In our study, the dura was expanded by reflecting its thicker outer layer and making longitudinal incisions in the inner layer. With time, CSF pulsations enable a remodeling to occur and the cisterna magna enlarges. In all the cases studied here, after approximately 10 months, the cisterna magna had nearly doubled in size. Radiological investigations revealed regression of the syringomyelia within 20 months. Imae *et al*. in their comparative study, have described four groups who underwent tonsillectomy with duraplasty, intra-arachnoidal lysis with duraplasty, duraplasty alone and delamination alone; the patients were followed up over a long period [36]. They found no significant difference among groups on the degree of reduction of syrinx whereas duraplasty was clearly less invasive compared to tonsillectomy or lysis. They suggested that duraplasty should be the primary surgical procedure for syringomyelia in CMI.

Alzate *et al*, in their series of 66 cases, stated that if no syringomyelic cavity existed, the dura splitting technique would have rendered better results, however, if the syringomyelic cavity was wide, drainage methods should be added [37]. Moreover, Munshi *et al*, suggested that if the cavity was not clear enough, results would be more satisfactory with bone decompression alone [32]. Limonadi *et al*, compared the duraplasty with dural splitting in pediatric patients with CMI and syringomyelia, and showed that dural splitting was more advantageous [35]. However, the dural splitting was performed without making internal dural incisions, as used in our study. They also showed that extra-arachnoidal osseodural decompression had fewer complications, a lower hospitalization period and lower costs than other techniques [35].

Williams showed that if the dura was left open, CSF circulation would improve [2]. However, Milhorat *et al*, advocated the opposite [14]. We believe that the dura mater can relax by dividing the outer layer from the inner layer and the result is even better if the inner layer is incised. The dura mater gets thinner while passing through foramen magnum to the spinal canal and its separation gets more difficult [28,29,31-33]. In our series, the dura splitting and incisions that we have made up to the cervical 2 were sufficient for a good result.

Concerning the reduction in size of the syringomyelic cavity, the percentage of success is evidently lower in our study than when prior dural and trans-arachnoidal approaches to therapy are performed. However, we believed that, even a moderate or no change on postoperative MR images may be regarded as a worthwhile result when accompanied by clinical recovery. Improved CSF circulation resulted in mild-moderate reduction in the syrinx cavity in 5 of 10 cases, whereas in the remaining patients the syrinx size remained stable on MR images. In these cases there was a significant improvement in the symptoms despite a persistent syrinx. Hence no further surgical procedure was advised to avoid even the minimal rate of risk of shunt complications or major intra-arachnoidal surgical complications.

Nakamura *et al*, have described before that in CMI, when dura mater histology is examined, there occurs a high production of collagen fibers [33]. Volumetric enlargement of the posterior fossa was not detected in our case. However, strong and thickened fibro-dural bands at the level of foramen magnum were always present and removing this tissue seemed to be the most important factor. This concurs with Nakamura *et al*. who suggested that the thickening of the dura mater may be causative factor of syringomyelia with CMI [33]. Our data help to verify the hypothesis that performing the simple posterior fossa bone decompression in patients with CMI can achieve comparable results to those obtained with more invasive surgical approaches. Indeed, in our study the percentage of good results is similar to that reported in the literature when more aggressive surgical approaches were used [38].

## Conclusion

Removal of the fibrous band and the outer dural layer, at level of foramen magnum, together with the incision of inner dural layer seems to be good technique for the treatment of syringomyelia in adult CMI patients. The advantages are short operation time, no need for duraplasty, sufficient posterior fossa decompression, absence of CSF fistulas as a result of extra arachnoidal surgery, and short duration of hospitalization. Hence this surgical technique may be superior compared to other techniques.

## Competing interests

The authors declare that they have no competing interests.

## Authors' contributions

KK(senior author) designed this study, performed the operations, drafted the manuscript, and conceived of the review. RT reviewed the clinical profile of the patients and was involved with the interpretation. TT carried out the literature search and drew the illustrations. YC performed the interpretation and collection of radiological data and evaluated preoperative and postoperative MRI findings. All authors read and approved the final version of the manuscript.
